# Current applications and challenges in large language models for patient care: a systematic review

**DOI:** 10.1038/s43856-024-00717-2

**Published:** 2025-01-21

**Authors:** Felix Busch, Lena Hoffmann, Christopher Rueger, Elon HC van Dijk, Rawen Kader, Esteban Ortiz-Prado, Marcus R. Makowski, Luca Saba, Martin Hadamitzky, Jakob Nikolas Kather, Daniel Truhn, Renato Cuocolo, Lisa C. Adams, Keno K. Bressem

**Affiliations:** 1 School of Medicine and Health, Department of Diagnostic and Interventional Radiology, Klinikum rechts der Isar, TUM University Hospital, Technical University of Munich, Munich, Germany; 2 Department of Neuroradiology, Charité – Universitätsmedizin Berlin, Corporate Member of Freie Universität Berlin and Humboldt Universität zu Berlin, Berlin, Germany; 3 Department of Ophthalmology, Leiden University Medical Center, Leiden, The Netherlands; 4 Department of Ophthalmology, Sir Charles Gairdner Hospital, Perth, Australia; 5 Division of Surgery and Interventional Sciences, University College London, London, United Kingdom; 6 One Health Research Group, Faculty of Health Science, Universidad de Las Américas, Quito, Ecuador; 7 Department of Radiology, Azienda Ospedaliero Universitaria (A.O.U.), Cagliari, Italy; 8 School of Medicine and Health, Institute for Cardiovascular Radiology and Nuclear Medicine, German Heart Center Munich, TUM University Hospital, Technical University of Munich, Munich, Germany; 9 Department of Medical Oncology, National Center for Tumor Diseases (NCT), Heidelberg University Hospital, Heidelberg, Germany; 10 Else Kroener Fresenius Center for Digital Health, Medical Faculty Carl Gustav Carus, Technical University Dresden, Dresden, Germany; 11 Department of Diagnostic and Interventional Radiology, University Hospital Aachen, Aachen, Germany; 12 Department of Medicine, Surgery and Dentistry, University of Salerno, Baronissi, Italy

**Keywords:** Health services, Diagnosis, Prognosis, Disease prevention, Therapeutics

## Abstract

**Background:**

The introduction of large language models (LLMs) into clinical practice promises to improve patient education and empowerment, thereby personalizing medical care and broadening access to medical knowledge. Despite the popularity of LLMs, there is a significant gap in systematized information on their use in patient care. Therefore, this systematic review aims to synthesize current applications and limitations of LLMs in patient care.

**Methods:**

We systematically searched 5 databases for qualitative, quantitative, and mixed methods articles on LLMs in patient care published between 2022 and 2023. From 4349 initial records, 89 studies across 29 medical specialties were included. Quality assessment was performed using the Mixed Methods Appraisal Tool 2018. A data-driven convergent synthesis approach was applied for thematic syntheses of LLM applications and limitations using free line-by-line coding in Dedoose.

**Results:**

We show that most studies investigate Generative Pre-trained Transformers (GPT)-3.5 (53.2%, *n* = 66 of 124 different LLMs examined) and GPT-4 (26.6%, *n* = 33/124) in answering medical questions, followed by patient information generation, including medical text summarization or translation, and clinical documentation. Our analysis delineates two primary domains of LLM limitations: design and output. Design limitations include 6 second-order and 12 third-order codes, such as lack of medical domain optimization, data transparency, and accessibility issues, while output limitations include 9 second-order and 32 third-order codes, for example, non-reproducibility, non-comprehensiveness, incorrectness, unsafety, and bias.

**Conclusions:**

This review systematically maps LLM applications and limitations in patient care, providing a foundational framework and taxonomy for their implementation and evaluation in healthcare settings.

## Plain Language Summary

Large language models (LLMs) are computer programs that can generate human-like text. They promise to improve patient education and expand access to medical information by helping patients better understand health conditions and treatment options. However, more information is needed about how these tools are used in patient care and the challenges they present. In this review, researchers analyzed 89 studies from 2022 to 2023 covering 29 medical specialties. These studies explored ways LLMs are used: for example, answering patient questions, summarizing or translating medical texts, and supporting clinical paperwork. While these tools show potential, the review highlights limitations. Many LLMs are not optimized for medical use, lack transparency about data use, and can be difficult for some users to access. Additionally, the text they generate may sometimes be inaccurate, incomplete, or biased, raising safety concerns.

## Abstract (web summary)

Busch et al. discuss large language models in patient healthcare. This systematic review analyzes current literature for utilization of these models and limitations of use and implementation.

## Introduction

Public and academic interest in large language models (LLMs) and their potential applications has increased substantially, especially since the release of OpenAI’s ChatGPT (Chat Generative Pre-trained Transformers) in November 2022^1–3^. One of the main reasons for their popularity is the remarkable ability to mimic human writing, a result of extensive training on massive amounts of text and reinforcement learning from human feedback^4^.

Since most LLMs are designed as general-purpose chatbots, recent research has focused on developing specialized models for the medical domain, such as Meditron or BioMistral, by enriching the training data of LLMs with medical knowledge^5,6^. However, this approach to fine-tuning LLMs requires significant computational resources that are not available to everyone and is also not applicable to closed-source LLMs, which are often the most powerful. Therefore, another approach to improve LLMs for biomedicine is to use techniques such as Retrieval-Augmented Generation (RAG)^7^. RAG allows information to be dynamically retrieved from medical databases during the model generation process, enriching the output with medical knowledge without the need to train the model.

LLMs hold great promise for improving the efficiency and accuracy of healthcare delivery, e.g., by extracting clinical information from electronic health records, summarizing, structuring, or explaining medical texts, streamlining administrative tasks in clinical practice, and enhancing medical research, quality control, and education^8–10^. In addition, LLMs have been shown to be versatile tools for supporting diagnosis or serving as prognostic models^11,12^.

However, despite the growing body of research and the clear potential of LLMs in healthcare, there is a gap in terms of systematized information towards their use in patient care (i.e., the use of LLMs by patients or their caregivers for disease management and support). In contrast to applications primarily aimed at healthcare professionals, LLMs in patient care could be used for education and empowerment by providing answers to medical questions and translating complex medical information into more accessible language^4,13^. Thereby, LLMs may promote personalized medicine and broaden access to medical knowledge, empowering patients to actively participate in their healthcare decisions.

To the best of our knowledge, there has been no evaluation of existing research to understand the scope of applications and identify limitations that may currently limit the successful integration of LLMs into clinical practice. This systematic review aims to analyze and synthesize the literature on LLMs in patient care, providing a systematic overview of 1) current applications and 2) challenges and limitations, with the purpose of establishing a foundational framework and taxonomy for the implementation and evaluation of LLMs in healthcare settings.

## Methods

This systematic review was pre-registered in the International Prospective Register of Systematic Reviews (PROSPERO) under the identifier CRD42024504542 before the start of the initial screening and was conducted according to the Preferred Reporting Items for Systematic Reviews and Meta-Analyzes (PRISMA) guidelines (see checklist in the Supplementary Dataset file 1)^14,15^.

### Eligibility criteria

We searched 5 databases, including the Web of Science, PubMed, Embase/Embase Classic, American for Computing Machinery (ACM) Digital Library, and Institute of Electrical and Electronics Engineers (IEEE) Xplore as of January 25, 2024, to identify qualitative, quantitative, and mixed methods studies published between January 1, 2022, and December 31, 2023, that examined the use of LLMs for patient care. LLMs for patient care were defined as any artificial neural network that follows a transformer architecture and can be used to generate and translate text and other content or perform other natural language processing tasks for the purpose of disease management and support (i.e., prevention, preclinical management, diagnosis, treatment, or prognosis) that could be directly directed to or used by patients. Articles had to be available in English and contain sufficient data for thematic synthesis (e.g., conference abstracts that did not provide sufficient information on study results were excluded). Given the recent surge in publications on LLMs such as ChatGPT, we allowed for the inclusion of preprints if no corresponding peer-reviewed article was available. Duplicate reports of the same study, non-human studies, and articles limited to technology development/performance evaluation, pharmacy, human genetics, epidemiology, psychology, psychosocial support, or behavioral assessment were excluded.

### Screening and data extraction

Initially, we conducted a preliminary search on PubMed and Google Scholar to define relevant search terms. The final search strategy included terms for LLMs, generative AI, and their applications in medicine, health services, clinical practices, medical treatments, and patient care (as detailed by database in the Supplementary Methods). After importing the bibliographic data into Rayyan and removing duplicates, LH and CR conducted an independent blind review of each article’s title and abstract^16^. Any article flagged as potentially eligible by either reviewer proceeded to the full-text evaluation stage. For this stage, LH and CR used a custom data extraction form created in Google Forms (available online)^17^ to collect all relevant data independently from the studies that met the inclusion criteria. Quality assessment was also performed independently for each article within this data extraction form, using the Mixed Methods Appraisal Tool (MMAT) 2018^18^. Disagreements at any stage of the review were resolved through discussion with the author FB. In cases of studies with incomplete data, we have tried to contact the corresponding authors for clarification or additional information.

### Data analysis

Due to the diversity of investigated outcomes and study designs we sought to include, a meta-analysis was not practical. Instead, a data-driven convergent synthesis approach was selected for thematic syntheses of LLM applications and limitations in patient care^19^. Following Thomas and Harden, FB coded each study’s numerical and textual data in Dedoose using free line-by-line coding^20,21^. Initial codes were then systematically categorized into descriptive and subsequently into analytic themes, incorporating new codes for emerging concepts within a hierarchical tree structure. Upon completion of the codebook, FB and LH reviewed each study to ensure consistent application of codes. Discrepancies were resolved through discussion with the author KKB, and the final codebook and analytical themes were discussed and refined in consultation with all contributing authors.

## Results

### Screening results

**Fig. 1:**
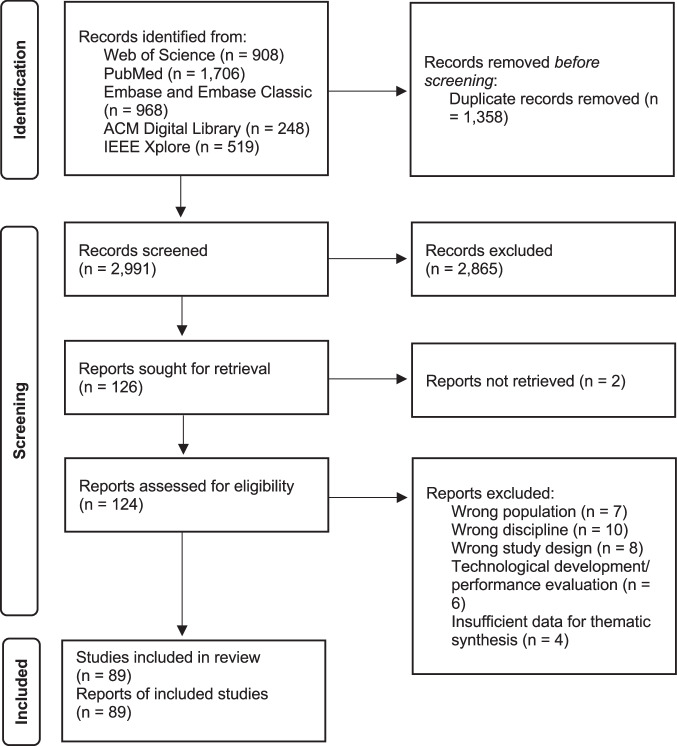
Preferred reporting items for systematic reviews and meta-analyzes (PRISMA) flow diagram. A total of 4349 reports were identified from Web of Science, PubMed, Embase/Embase Classic, ACM Digital Library, and IEEE Xplore. After excluding 1358 duplicates, 2991 underwent initial screening and 126 were deemed suitable for potential inclusion and underwent full-text screening. Two articles could not be retrieved because the authors or the corresponding title and abstract could not be identified online. After full text screening, 35 articles were excluded and 89 articles were included in the final review.

Of the 4349 reports identified, 2991 underwent initial screening, and 126 were deemed suitable for potential inclusion and underwent full-text screening. Two articles could not be retrieved because the authors or the corresponding title and abstract could not be identified online. Following full-text screening, 35 articles were excluded, and 89 articles were included in the final review. Most studies were excluded because they targeted the wrong discipline (*n* = 10/35, 28.6%) or population (*n* = 7/35, 20%) or were not original research (*n* = 8/35, 22.9%) (see Supplementary Dataset file 2). For example, we evaluated a study that focused on classifying physician notes to identify patients without active bleeding who were appropriate candidates for thromboembolism prophylaxis^22^. Although the classification tasks may lead to patient treatment, the primary outcome was informing clinicians rather than directly forwarding this information to patients. We also reviewed a study assessing the accuracy and completeness of several LLMs when answering Methotrexate-related questions^23^. This study was excluded because it focused solely on the pharmacological treatment of rheumatic disease. For a detailed breakdown of the inclusion and exclusion process at each stage, please refer to the PRISMA flowchart in Fig. 1.

### Characteristics of included studies

Supplementary Dataset file 3 summarizes the characteristics of the analyzed studies, including their setting, results, and conclusions. One study (*n* = 1/89, 1.1%) was published in 2022^24^, 84 (*n* = 84/89, 94.4%) in 2023^13,25–107^, and 4 (*n* = 4/89, 4.5%) in 2024^108–111^ (all of which were peer-reviewed publications of preprints published in 2023). Most studies were quantitative non-randomized (*n* = 84/89, 94.4%)^13,25–27,29–101,103,104,106,107,109–111^, 4 (*n* = 4/89, 4.5%)^28,102,105,108^ had a qualitative study design, and one (*n* = 1/89, 1.1%)^24^ was quantitative randomized according to the MMAT 2018 criteria. However, the LLM outputs were often first analyzed quantitatively but followed by a qualitative analysis of certain responses. Therefore, if the primary outcome was quantitative, we considered the study design to be quantitative rather than mixed methods, resulting in the inclusion of zero mixed methods studies. The quality of the included studies was mixed (see Supplementary Dataset file 4). The authors were primarily affiliated with institutions in the United States (*n* = 47 of 122 different countries identified per publication, 38.5%), followed by Germany (*n* = 11/122, 9%), Turkey (*n* = 7/122, 5.7%), the United Kingdom (*n* = 6/122, 4.9%), China/Australia/Italy (*n* = 5/122, 4.1%, respectively), and 24 (*n* = 36/122, 29.5%) other countries. Most studies examined one or more applications based on the GPT-3.5 architecture (*n* = 66 of 124 different LLMs examined per study, 53.2%)^13,26–29,31–34,36–40,42–49,52–54,56–61,63,65–67,71,72,74,75,77,78,81–89,91,92,94,95,97–100,102–104,106–109,111^, followed by GPT-4 (*n* = 33/124, 26.6%)^13,25,27,29,30,34–36,41,43,50,51,54,55,58,61,64,68–70,74,76,79–81,83,87,89,90,93,96,98,99,101,105^, Bard (*n* = 10/124, 8.1%; now known as Gemini)^33,48,49,55,73,74,80,87,94,99^, Bing Chat (*n* = 7/124, 5.7%; now Microsoft Copilot)^49,51,55,73,94,99,110^, and other applications based on Bidirectional Encoder Representations from Transformers (BERT; *n* = 4/124, 3.2%)^13,83,84^, Large Language Model Meta-AI (LLaMA; *n* = 3/124, 2.4%)^55^, or Claude by Anthropic (*n* = 1/124, 0.8%)^55^. The majority of applications were primarily targeted at patients (*n* = 64 of 89 included studies, 73%)^24,25,29,32,34–39,41–43,45–48,52–54,56–60,62,63,65,66,68–71,73–75,77–80,85–95,97,99,100,102–111^ or both patients and caregivers (*n* = 25/89, 27%)^13,26–28,30,31,33,40,44,49–51,55,61,64,67,72,76,81–84,96,98,101^. Information about conflicts of interest and funding was not explicitly stated in 23 (*n* = 23/89, 25.8%) studies, while 48 (*n* = 48/89, 53.9%) reported that there were no conflicts of interest or funding. A total of 18 (*n* = 18/89, 20.2%) studies reported the presence of conflicts of interest and funding^13,24,38,40,54,58,59,67,69–71,74,80,84,96,103,105,111^. Most studies did not report information about the institutional review board (IRB) approval (*n* = 55/89, 61.8%) or deemed IRB approval unnecessary (*n* = 28/89, 31.5%). Six studies obtained IRB approval (*n* = 6/89, 6.7%)^52,82,84–86,92^.

### Applications of large language models

**Fig. 2:**
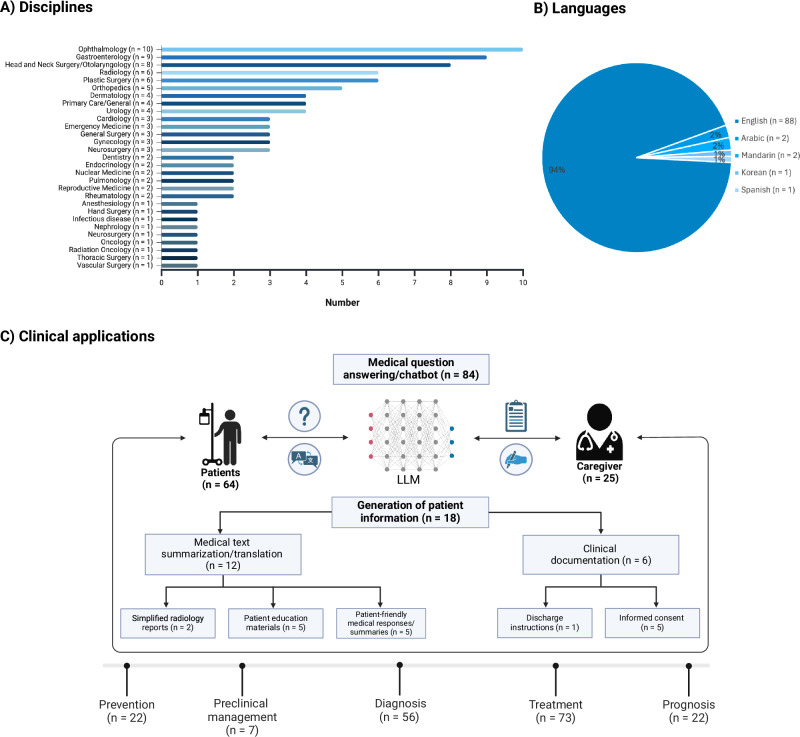
Schematic illustration of the identified disciplines, languages, and clinical concepts of large language models (LLMs) applications in patient care. **A** Column plot showing the distribution of medical specialties in which LLMs have been tested for patient care. **B** Pie chart illustrating the distribution of languages in which LLMs have been tested. **C** Schematic representation of the concepts identified for the application of LLMs in patient care.

An overview of the presence of codes for each study is provided in the Supplementary Dataset file 3. The majority of articles investigated the use and feasibility of LLMs as medical chatbots (*n* = 84/89, 94.4%)^13,24–62,64–66,68,69,71–96,98–111^, while fewer reports additionally or exclusively focused on the generation of patient information (*n* = 18/89, 20.2%)^24,31,43,48,49,57,59,62,67,79,88–91,97,102,106,107^, including clinical documentation such as informed consent forms (*n* = 5/89, 5.6%)^43,67,91,97,102^ and discharge instructions (*n* = 1/89, 1.1%)^31^, or translation/summarization tasks of medical texts (*n* = 5/89, 5.6%)^24,49,57,79,89^, creation of patient education materials (*n* = 5/89, 5.6%)^48,62,90,106,107^, and simplification of radiology reports (*n* = 2/89, 2.3%)^59,88^. Most reports evaluated LLMs in English (*n* = 88/89, 98.9%)^13,24–103,105–111^, followed by Arabic (*n* = 2/84, 2.3%)^32,104^, Mandarin (*n* = 2/84, 2.3%)^36,75^, and Korean or Spanish (*n* = 1/89, 1.1%, respectively)^75^. The top-five specialties studied were ophthalmology (*n* = 10/89, 11.2%)^37,40,48,51,65,74,97,98,100,101^, gastroenterology (*n* = 9/89, 10.1%)^25,32,34,36,39,61,62,72,96^, head and neck surgery/otolaryngology (*n* = 8/89, 9%)^35,42,56,64,66,76,78,79^, and radiology^59,70,88–90,110^ or plastic surgery^45,47,49,102,107,108^ (*n* = 6/89, 6.7%, respectively). A schematic illustration of the identified concepts of LLM applications in patient care is shown in Fig. 2.

### Limitations of large language models

**Fig. 3:**
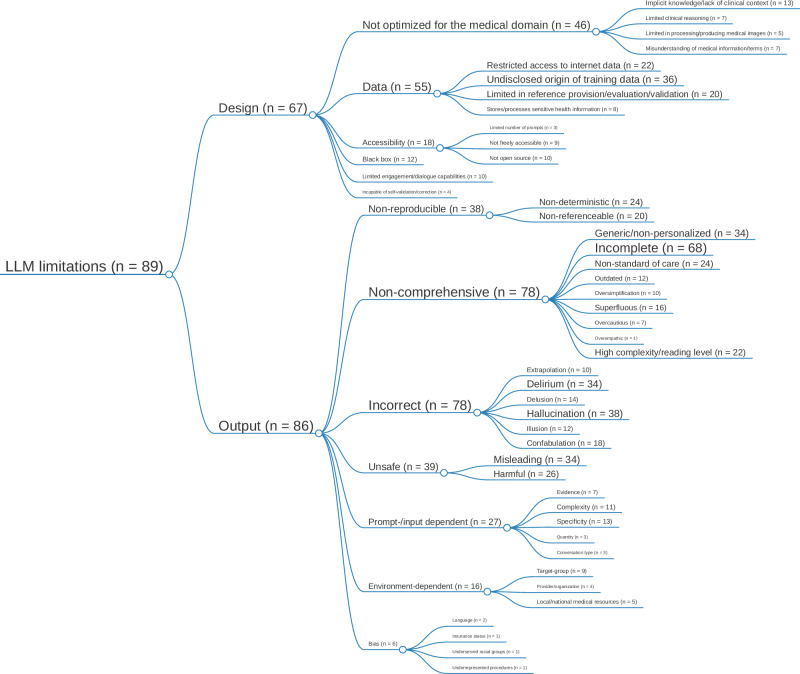
Illustration of the hierarchical tree structure for the thematic synthesis of large language model (LLM) limitations in patient care, including the presence of codes for each concept. The font size of each concept is shown in proportion to its frequency in the studies analyzed. Our analysis delineates two primary domains of LLM limitations: design and output. Design limitations included 6 second-order and 12 third-order codes, while output limitations included 9 second-order and 32 third-order codes.

The thematic synthesis of limitations resulted in two main concepts: one related to design limitations and one related to output. Figure 3 illustrates the hierarchical tree structure and quantity of the codes derived from the thematic synthesis of limitations. Supplementary Dataset file 5 provides an overview of the taxonomy of all identified limitation concepts, including their description and examples.

#### Design limitations

In terms of design limitations, many authors noted the limitation that LLMs are not optimized for medical use (*n* = 46/89, 51.7%)^13,26,28,34,35,37–39,46,49,50,54–59,61,62,65,66,68,70,71,79–81,83–85,88,91,93–98,100–107,109^, including implicit knowledge/lack of clinical context (*n* = 13/89, 14.6%)^28,39,46,66,71,79,81,83–85,98,103^, limitations in clinical reasoning (*n* = 7/89, 7.9%)^55,84,95,102–105^, limitations in medical image processing/production (*n* = 5/89, 5.6%)^37,55,91,106,107^, and misunderstanding of medical information and terms by the model (*n* = 7/89, 7.9%)^28,38,39,59,62,65,97^. In addition, data-related limitations were identified, including limited access to data on the internet (*n* = 22/89, 24.7%)^38,39,41,43,54–57,59,60,64,76,79,82–84,88,91,94,96,104,109^, the undisclosed origin of training data (*n* = 36/89, 40.5%)^25,26,29,30,32,34,36,37,40,46,47,50,51,53–60,64,65,70,71,76,82,83,91,94–96,101,105,109^, limitations in providing, evaluating, and validating references (*n* = 20/89, 22.5%)^45,49,54–57,65,71,73,76,80,83,85,91,94,96,98,101,103,105^, and storage/processing of sensitive health information (*n* = 8/89, 9%)^13,34,46,55,62,76,83,109^. Further second-order concepts included black-box algorithms, i.e., non-explainable AI (*n* = 12/89, 13.5%)^27,36,55,57,65,73,76,83,91,94,103,105^, limited engagement and dialog capabilities (*n* = 10/89, 11.2%)^13,27,28,37,38,51,56,66,95,103^, and the inability of self-validation and correction (*n* = 4/89, 4.5%)^61,73,74,107^.

#### Output limitations

The evaluation of limitations in output data yielded 7 second-order codes concerning the non-reproducibility (*n* = 38/89, 42.7%)^28,29,34,38,39,41,43,45,46,49,54–61,64,65,71–73,76,80,82,83,85,90,91,94,96,98,99,101,103–105^, non-comprehensiveness (*n* = 78/89, 87.6%)^13,25,26,28–30,32–44,46,48–62,64,65,67–79,81–98,100,102–107,109–111^, incorrectness (*n* = 78/89, 87.6%)^13,25–44,46,49–52,54–62,64–66,69–79,81–85,87–107,109–111^, (un-)safeness (*n* = 39/89, 43.8%)^28,30,35,37,39,40,42–44,46,50,51,57–60,62,64,65,69,70,73,74,76,78–80,82,84,85,91,94,95,98–100,105,106,109^, bias (*n* = 6/89, 6.7%)^26,32,34,36,66,103^, and the dependence of the quality of output on the prompt-/input provided (*n* = 27/89, 30.3%)^26–28,34,38,41,44,46,51,52,56,68–72,74,76,78,79,81–83,90,94,95,100,101^ or the environment (*n* = 16/89, 18%)^13,34,46,49–51,54,58,60,72,73,88,90,93,97,109^.

##### Non-reproducibility

For non-reproducibility, key concepts included the non-deterministic nature of the output, e.g., due to inconsistent results across multiple iterations (*n* = 34/89, 38.2%)^28,29,34,38,39,41,43,46,58–61,72,76,82,90,94,98,99,101,103,104^ and the inability to provide reliable references (*n* = 20/89, 22.5%)^45,49,54–57,65,71,73,76,80,83,85,91,94,96,98,101,103,105^.

##### Non-comprehensiveness

Non-comprehensiveness included nine concepts related to generic/non-personalized output (*n* = 34/89, 38.2%)^13,28,30,34,37,38,41,43,49,51,56,57,59,61,65,70,77,79,81,84–86,90,94,95,100,102–107,110^, incompleteness of output (*n* = 68/89, 76.4%)^13,25,26,28–30,32,34–39,41–44,46,49–52,55–62,64,65,67–69,72–77,79,81–86,89–98,100,102–107,109–111^, provision of information that is not standard of care (*n* = 24/89, 27%)^28,40,43,46,49,50,54,57,58,65,69,72,73,77,78,81,85,91,94,98,100,103,107,111^ and/or outdated (*n* = 12/89, 13.5%)^13,25,32,34,38,41,43,44,49,54,83,84^, and production of oversimplified (*n* = 10/89, 11.2%)^38,46,49,54,59,79,84,85,103^, superfluous (*n* = 16/89, 18%)^13,28,34,38,46,62,72,79,86,90,94,97,100,106,107^, overcautious (*n* = 7/89, 7.9%)^13,28,37,51,70,103,110^, overempathic (*n* = 1/89, 1.1%)^13^, or output with inappropriate complexity/reading level for patients (*n* = 22/89, 24.7%)^13,34,42,48,50,51,53,55,56,67,71,78,79,85,87,88,90,93,106,107,109,110^.

##### Incorrectness

For incorrectness, we identified 6 key concepts. Some of the incorrect information could be attributed to what is commonly known as hallucination (*n* = 38/89, 42.7%)^25,28,32,33,35–38,40–44,49–51,57–60,65,73,74,76,77,81,83,85,91,94,96–98,100,103,106,107,109^, i.e., the creation of entirely fictitious or false information that has no basis in the input provided or in reality (e.g., “You may be asked to avoid eating or drinking for a few hours before the scan” for a bone scan). Other instances of misinformation were more appropriately classified under alternative concepts of the original psychiatric analogy, as described in detail by Currie et al.^43,112,113^. These include illusion (*n* = 12/89, 13.5%)^28,36,38,43,57,59,77,78,85,88,94,105^, which is characterized by the generation of deceptive perceptions or the distortion of information by conflating similar but separate concepts (e.g., suggesting that MRI-type sounds might be experienced during standard nuclear medicine imaging), delirium (*n* = 34/89, 38.2%)^13,26,28,30,37,43,50,58,59,61,65,70,72–75,77,79,81–85,90–92,94,95,98,102,103,107,109,110^, which indicates significant gaps in vital information, resulting in a fragmented or confused understanding of a subject (e.g., omission of crucial information about caffeine cessation for stress myocardial perfusion scans), extrapolation (*n* = 11/89, 12.4%)^43,59,65,78,81,91,94,106,107,110^, which involves applying general knowledge or patterns to specific situations where they are inapplicable (e.g., advice about injection-site discomfort that is more typical of CT contrast administration), delusion (*n* = 14/89, 15.7%)^28,30,43,50,59,65,69,73,74,78,81,94,103,111^, a fixed, false belief despite contradictory evidence (e.g., inaccurate waiting times for the thyroid scan), and confabulation (*n* = 18/89, 20.2%)^25,28,36–38,40,46,59,62,65,71,77–79,94,103,107^, i.e., filling in memory or knowledge gaps with plausible but invented information (e.g., “You should drink plenty of fluids to help flush the radioactive material from your body” for a biliary system–excreted radiopharmaceutical).

##### Safety and bias

Many studies rated the generated output as unsafe, including misleading (*n* = 34/89, 38.2%)^28,30,35,43,44,46,50,51,57–60,62,64,65,69,73,74,76,78–80,82,84,85,94,95,98–100,105,106,109^ or even harmful content (*n* = 26/89, 29.2%)^28,30,37,39,40,42,43,50,51,58–60,70,73,74,76,79,84,85,91,94,95,98–100,109^.

A minority of reports identified biases in the output, which were related to language (*n* = 2/89, 2.3%)^32,36^, insurance status^103^, underserved racial groups^26^, or underrepresented procedures^34^ (*n* = 1/89, 1.1%, each).

##### Dependence on input and environment

Many authors suggested that performance was related to the prompting/input provided or the environment, i.e., depending on the evidence (*n* = 7/89, 7.9%)^52,68,69,71,81,82,95^, complexity (*n* = 11/89, 12.4%)^28,34,44,46,70,74,76,79,94,102^, specificity (*n* = 13/89, 14.6%)^27,38,41,56,70,72,74,76,78,81,95,100,101^, quantity (*n* = 3/89, 3.4%)^26,52,74^ of the input, type of conversation (*n* = 3/89, 3.4%)^27,51,90^, or the appropriateness of the output related to the target group (*n* = 9/89, 10.1%)^46,49,51,54,72,90,93,97,109^, provider/organization (*n* = 4/89, 4.5%)^13,50,60,88^, and local/national medical resources (*n* = 5/89, 5.6%)^34,50,58,60,73^.

## Discussion

In this systematic review, we synthesized the current applications and limitations of LLMs in patient care, incorporating a broad analysis across 29 medical specialties and highlighting key limitations in LLM design and output, providing a comprehensive framework and taxonomy for describing and categorizing limitations that may arise when using LLMs in healthcare settings.

Most articles examined the use of LLMs based on the GPT-3.5 or GPT-4 architecture for answering medical questions, followed by the generation of patient information, including medical text summarization or translation and clinical documentation. The conceptual synthesis of LLM limitations revealed two key concepts: the first related to design, including 6 second-order and 12 third-order codes, and the second related to output, including 9 second-order and 32 third-order codes. By systematically categorizing the limitations of LLMs in clinical settings, our taxonomy aims to provide healthcare professionals and developers with a framework for assessing potential risks associated with the use of LLMs in patient care. In addition, our work highlights key areas for improvement in the development of LLMs and aims to enable clinicians to make more informed decisions by understanding the limitations inherent in the design and output, thereby supporting the establishment of best practices for LLM use in clinical settings.

Although many LLMs have been developed specifically for the biomedical domain in recent years, we found that ChatGPT has been a disruptor in the medical literature on LLMs, with GPT-3.5 and GPT-4 accounting for almost 80% of the LLMs examined in this systematic review. While it was not possible to conduct a meta-analysis of the performance on medical tasks, many authors provided a positive outlook towards the integration of LLMs into clinical practice. However, we have conceptualized several key limitations in the design and output of LLMs, some of the most prevalent in our systematic review are briefly discussed in the following paragraphs.

The majority of studies (*n* = 55/89) reported limitations that were conceptualized as related to the underlying data of the LLMs studied. Especially the use of proprietary models such as ChatGPT in the biomedical field was a concern in many of the studies analyzed, mainly because of the lack of training data transparency (third-order code: undisclosed origin of training data). In practice, it is widely recognized that limited access to the underlying algorithms, training data, and data processing and storage mechanisms of LLMs is a significant barrier to their application in healthcare^114^. This opacity makes it difficult for healthcare professionals to fully understand how these models function, assess their reliability, or ensure compliance with local medical standards and regulations. Consequently, the use of such models in healthcare settings can be problematic, and the need to recognize and correct potential limitations in the outputs of such models is paramount.

Moreover, integrating proprietary models into clinical practice introduces a vulnerability to performance changes that occur with model updates^115^. As these models are updated by their developers, functionalities that healthcare providers rely on may be altered or broken, potentially leading to harmful outcomes for patients, which was also conceptualized in our study under output limitations (second-order code: unsafe; third-order codes: misleading/harmful). This unpredictability is a serious concern in the biomedical field, where consistency and reliability are crucial. Notably, the unpredictability of LLMs was another concept of output limitations in our systematic review (second-order code: non-reproducible; third-order codes: non-deterministic/non-referenceable).

As a result, open-source models such as BioMistral may offer a viable solution^6^. Such open source models not only offer more transparency, as their algorithms and training data are accessible but can also be adapted locally. However, given the limited number of articles on open-source LLMs in our review, we strongly encourage future studies investigating the applicability of open-source LLMs in patient care.

About half of the studies analyzed reported limitations related to LLMs not being optimized for the medical domain. One possible solution to this limitation may be to provide medical knowledge during inference using RAG^116^. However, even when trained for general purposes, ChatGPT has previously been shown to pass the United States Medical Licensing Examination (USMLE), the German State Examination in Medicine, or even a radiology board-style examination without images^117–120^. Although outperformed on specific tasks by specialized medical LLMs, such as Google’s MedPaLM-2, this suggests that general-purpose LLMs can comprehend complex medical literature and case scenarios to a degree that meets professional standards^121^. Furthermore, given the large amounts of data on which proprietary models such as ChatGPT are trained, it is not unlikely that they have been exposed to more medical data overall than smaller specialized models despite being generalist models. Notably, a recent study even suggested that fine-tuning LLMs on biomedical data does not improve performance compared to their general-purpose counterparts^122^.

It should also be noted that passing these exams does not equate to the practical competence required of a healthcare provider, which was also a limitation identified in our review (third-order codes: implicit knowledge/lack of clinical context; limited clinical reasoning; misunderstanding of medical information/terms; limited in processing/producing medical images)^123^. In addition, reliance on exam-based assessments carries a significant risk of bias. For example, if the exam questions or similar variants are publicly available and, thus, may be present in the training data, the LLM does not demonstrate any knowledge outside of training data memorization^124^. In fact, these types of tests can be misleading in estimating the model’s true abilities in terms of comprehension or analytical skills.

The non-reproducibility of LLM output, as conceptualized in 38 studies, highlights key challenges in ensuring consistency and determinism in LLM-generated results. One major issue is the inherent stochasticity in the models’ architecture, particularly in transformer-based models, which utilize probabilistic techniques during inference (e.g., beam search or temperature sampling)^125^. This non-determinism can lead to different outputs for the same input, making it difficult to replicate results exactly across different instances or even across models with identical training data. Further external factors contributing to non-reproducibility, such as variations in hardware, software versions, or context windows, complicate the assurance of reproducibility^126^. As the reproducibility of results is a central principle in medical practice, our concepts highlight the need for more standardized protocols, improved documentation of model configurations, the examination of non-determinism for evaluation purposes, and further research on how robust results can be achieved before implementing LLMs in real-world clinical practice. Interestingly, Ouyang et al. reported that only a minority of studies take non-determinism into account in their experimental evaluation when using ChatGPT for code generation, suggesting that this limitation is also prevalent and overlooked in other domains of LLM use^125^.

The concept of non-comprehensiveness was prevalent in almost 90% of the studies analyzed (*n* = 78/89). For this concept, the majority of third-order codes were related to LLM outputs that were incomplete. This issue is particularly significant when considering the application of LLMs in medical tasks such as clinical decision support or diagnosis, where incomplete or partial results can have serious consequences. In clinical practice, missing key information could lead to suboptimal patient outcomes, incorrect diagnoses, or improper treatment recommendations. For instance, an incomplete therapy suggestion could render the entire treatment plan insufficient, potentially resulting in harm to the patient. Given the potential of using LLMs in medical decision-making, these limitations underscore the necessity for expert supervision and validation of LLM outputs depending on their application. While LLMs used as chatbots for general patient inquiries may not require consistent human oversight, using LLMs for treatment advice would require consistent validation to ensure that incomplete information does not lead to adverse outcomes. Depending on their application, the same problem arises when the LLM generates generic or non-personalized information, which was another third-order code identified. The generation of content with high complexity and an inappropriate reading level, which was above the American Medical Association (AMA) recommended 6th-grade reading level in almost all of the 22 studies that analyzed the complexity level of the output, may further limit its usefulness for patient information^127^. Again, the best solution to the lack of comprehensiveness in clinical practice so far seems to be human oversight.

Incorrectness, alongside non-comprehensiveness (as above), was the most common second-order code, identified in about 90% of studies (n = 78/89). In our conceptual synthesis of incorrect results, we followed the taxonomy of Currie et al. to classify incorrect outputs more precisely into illusions, delusions, delirium, confabulation, and extrapolation, thus proposing a framework for a more precise and structured error classification to improve the characterization of incorrect outputs and enabling more detailed performance comparisons with other research^43,112,113^.

Many studies currently refer to all non-factual LLM results as “hallucinations.” However, this generalization fails to capture the complexity of errors when considering the original psychiatric analogy. Simply classifying errors as hallucinations restricts their description to invented information, overlooking errors that, for example, omit critical information and leading to fragmented or confused understanding (third-order code: delirium). Notably, the third-order code “delirium” was observed in nearly as many studies as the third-order code “hallucination.” However, a non-detailed classification of incorrect results can affect not only the comparability of research findings but also has implications for clinical practice. While hallucinations (for example, fabricating instructions like “You may need to fast before a bone scan.”) may not always have serious consequences, errors classified as delirium—such as omitting crucial details like caffeine cessation before a stress myocardial perfusion scan—would always result in undesired outcomes (in the here presented example, most likely in repeating or postponing the examination). As a result, our review advocates for a more detailed classification of incorrect results in order to increase the qualitative comparability of incorrect LLM outputs and, ultimately, the relevance and implications of these results for clinical practice.

The conceptualization of unsafeness in 39 of 89 studies presents a significant concern when considering the integration of LLMs into medical practice. In the field of medicine, any tool or intervention that could lead to misleading or harmful outcomes must be critically assessed, as the potential for patient harm is high^128,129^. Such tools are generally only accepted when the benefits clearly outweigh the risks, and even then, informed consent from the affected individual is essential^130^. While informed consent might ensure that patients understand the risks involved and are able to make an educated decision about their care, which could be obtained, for example, in the form of a disclaimer before using the LLM, studies suggest that even when obtaining informed consent the patient understanding increases not significantly^131^. In the case of disclaimers, there might also be the risk that these are accepted without proper reading or understanding^132^. The practicality of informed consent once LLMs are deeply integrated into clinical workflows also remains an issue, as it is when patients no longer have the ability to opt out, such as in the case of serious illness. In any case, the finding that nearly half of the studies reported limitations related to LLM unsafeness suggests that LLMs are not yet reliable enough for autonomous medical use, and there is a critical need for safety measures and regulatory and human oversight to prevent adverse consequences in medical contexts^133^.

Further second-order concepts suggested that the output is influenced by the input or environment in which it is expressed. In fact, LLMs can be highly dependent on the quality and specificity of input, making their output prone to errors when faced with vague or incomplete information^134–136^. Again, this poses significant risks in patient care, where incorrect outputs can lead to adverse outcomes, such as inappropriate triage or treatment. For example, in our review, eleven studies reported a decrease in performance with increasing complexity of the input, which can have implications in clinical practice, such as failing to consider multifactorial medical issues like comorbidities, thus compromising the quality of care for those patients.

We found that the environment also influences the appropriateness of LLM outputs in medical settings. Models may recommend treatments that are inappropriate for certain patient populations, such as offering adult care protocols for pediatric patients or suggesting therapies that are not available in certain regions. This also raises ethical concerns, particularly in resource-constrained settings, where making inappropriate or inaccessible recommendations may reinforce existing inequalities and lead to uncomfortable situations for both the healthcare provider and the patient^137^. One solution may be to provide adequate training to LLM users, or in our scenario, to patients, on how to present input to the model to achieve the best results. Another solution is to train or fine-tune the model to the environment in which it will be used. For example, if the LLM is trained on the standard operating procedure for handling patients with major adverse cardiovascular events in a particular hospital, it is more likely to recommend the adequate procedure in this setting than when it is trained on worldwide data from an unknown time frame, where there is a chance that it will suggest non-standard care that may only be relevant in other countries where most of the training data is coming from, or even provides outdated information (which is another third-order code that was conceptualized under non-comprehensiveness) if it is trained on data that is not current.

Ultimately, only six studies have identified biases in their results, for example, reflecting the unequal representation of certain content or the biases inherent in human-generated text in the training data^138^. Here, we conceptualized the results of studies that identified bias in their analysis and not only mentioned bias as a theoretical limitation. Thus, these results may indicate that the implemented safeguards are effective. On the other hand, identifying bias was not the primary outcome of most studies, and not much is known about the technology and developer policies of proprietary LLMs. Moreover, previous work has shown that automated jailbreak generation is possible across various commercial LLM chatbots^139^. In the end, LLMs are trained on large datasets that inevitably contain biases—such as gender, racial, or cultural biases—embedded in the text^140^. These biases can be amplified or reflected by the models, leading to unfair or harmful outputs. Despite the use of various mitigation techniques, such as debiasing algorithms, curating balanced datasets, or incorporating fairness-focused training objectives, eliminating bias entirely is a persistent challenge^141–143^. This is because LLMs learn patterns from their training data, and human biases are inherently present in much of the data they consume. Moreover, the biases introduced or reinforced by LLM are not always obvious, making them more difficult to detect and correct, which may have contributed to the comparatively low number of studies that reported any bias in their results. Notably, subtle biases, such as those related to linguistic connotations, regional dialects, or implicit associations, can be especially insidious and difficult to eliminate through technical safeguards^144^. Therefore, the results of our review may encourage future studies to more explicitly examine the biases inherent in LLMs when used for medical tasks and how such biases could be mitigated.

Our findings raise a key question when applying LLMs to the medical domain: how can we entrust our patients to LLMs if they are neither reliable nor transparent? Given that models like ChatGPT are already publicly accessible and widely used, patients may already refer to them for medical questions in much the same way they use Google Search, making concerns about their early adoption somewhat academic^145^.

In addition to the advances in the development of LLMs and the focus on open source, adopting appropriate security measures to prevent the identified LLM limitations in clinical practice out-of-the-box will become increasingly important. For example, strategies to ensure LLM security and privacy can include continuous monitoring for new vulnerabilities, implementing input validation, conducting regular audits of training data, and using secure data pipelines^146^. Additionally, data anonymization, encryption, access controls, and regular security updates are essential to prevent data leakage, model theft, and privacy breaches.

Moreover, expert oversight of the final LLM output could mitigate any remaining risks in the last instance, ensuring that erroneous or inappropriate suggestions are identified and corrected before they can impact patient care. Recently, efforts have been made in this direction by adopting the widely recognized Physician Documentation Quality Instrument (PDQI-9) for the assessment of AI transcripts and clinical summaries^147^. However, whether ongoing human oversight and validation of LLM-generated content is feasible and can reduce the likelihood of adverse outcomes remains the subject of further research at this early stage of LLM deployment in healthcare.

Another important factor for the successful clinical implementation of LLMs in patient care could be patient acceptance, which was not assessed in any of the studies analyzed. The growing use of LLMs in healthcare might be perceived as a reduction in the interpersonal relationship between healthcare professionals and patients, potentially leading to a sense of dehumanization in medicine^148^. Therefore, to promote a positive reception of AI tools among patients, incorporating their perspectives already during the AI development and implementation process could be key^149^. Eventually, patient perspectives are already considered in AI regulatory frameworks, such as in the European Union AI Act, which came into force in August 2024^150^. The associated challenges faced by generative AI and LLM, for example, in terms of training data transparency and validation of non-deterministic output, will show which approaches the companies will take to bring these models into compliance with the law^151^. How the notified bodies interpret and enforce the law in practice will likely be decisive for the further development of LLMs in the biomedical sector.

Our study has limitations. First, our review focused on LLM applications and limitations in patient care, thus excluding research directed at clinicians only. Future studies may extend our synthesis approach to LLM applications that explicitly focus on healthcare professionals. Second, while it was not possible to conduct a meta-analysis of LLM performance due to the different study designs and evaluation methods used, this will be an important area for future work as the field of LLM research in clinical settings continues to evolve. Third, there is a risk that potentially eligible studies were not included in our analysis if they were not present in the 5 databases reviewed or were not available in English. However, we screened nearly 3,000 articles in total and systematically analyzed 89 articles, providing a comprehensive overview of the current state of LLMs in patient care, even if some articles could have been missed. With our chosen cut-off date of January 2022, there is also a risk of missing relevant publications on predecessor LLM models, such as GPT-3, which was introduced in 2020. However, as our review focused on current LLM applications and limitations, it seemed most beneficial to include only recent publications from the last two years on the most advanced models, especially when considering that ChatGPT was first made available in November 2022. Finally, the rapid development and advancement of LLMs make it difficult to keep this systematic review up to date. For example, Gemini 1.5 Pro was published in February 2024, and corresponding articles are not included in this review, which synthesized articles from 2022 to 2023. This also has implications for our introduced taxonomy of LLM limitations, as new limitations may emerge as models evolve, and previous limitations may become less relevant or even obsolete. For example, our taxonomy identifies “limited access to internet data” as a limitation; however, with the introduction of web browsing capabilities for GPT-4 in May 2023, this particular limitation no longer applies to that model. Given these ongoing developments, we strongly encourage future studies to test, update, and extend our taxonomy to ensure that it remains a relevant tool for categorizing LLM limitations in clinical and other high-stakes applications.

## Supplementary information

Supplementary Methods

Description of Additional Supplementary Files

Supplementary Dataset 1

Supplementary Dataset 2

Supplementary Dataset 3

Supplementary Dataset 4

Supplementary Dataset 5

## Data Availability

All data generated or analyzed during this study, including source data, are included in this published article and its supplementary information files. To provide a more intuitive overview and allow readers to filter through the collected study data and codes, we have provided the Supplementary Dataset files in the form of Excel spreadsheets.
