# Supplementary material for: Current applications and challenges in large language models for patient care: a systematic review
Source: Commun Med (Lond). 2025 Jan 21;5:26. doi: 10.1038/s43856-024-00717-2 (PMC11751060; doi:10.1038/s43856-024-00717-2)
Supplement: Supplementary file 1 — Supplementary Methods [file 43856_2024_717_MOESM1_ESM.pdf]

Supplementary Methods

Database search strategy

1. Web of Science
TS=("large language model" OR "LLM" OR "GPT" OR "BERT" OR "transformer model" OR "generative model" OR "generative AI" OR "generative artificial intelligence" OR "generative pre-trained transformer" OR "bidirectional encoder representations from transformers" OR "AI language model") AND TS=(medical practice OR healthcare OR clinical practice OR medicine OR medical treatment OR patient) AND PY=(2022-2023) AND LA=(English) NOT DT=(Review)

2. PubMed
("large language model" OR "LLM" OR "GPT" OR "BERT" OR "transformer model" OR "generative model" OR "generative AI" OR "generative artificial intelligence" OR "generative pre-trained transformer" OR "bidirectional encoder representations from transformers" OR "AI language model") AND (medical practice OR healthcare OR clinical practice OR medicine OR medical treatment OR patient) NOT Review[Publication Type] 2022:2023 [dp] English[la]

3. Embase and Embase Classic
Limit to (english language and yr="2022 - 2023")
("large language model" OR "LLM" OR "GPT" OR "BERT" OR "transformer model" OR "generative model" OR "generative AI" OR "generative artificial intelligence" OR "generative pre-trained transformer" OR "bidirectional encoder representations from transformers" OR "AI language model").mp. AND (medical practice OR healthcare OR clinical practice OR medicine OR medical treatment OR patient).mp. NOT "Review".pt.

4. ACM Digital Library
[[Title: "large language model"] OR [Abstract: "large language model"] OR [Title: "llm"] OR [Abstract: "llm"] OR [Title: "gpt"] OR [Abstract: "gpt"] OR [Title: "bert"] OR [Abstract: "bert"] OR [Title: "transformer model"] OR [Abstract: "transformer model"] OR [Title: "generative model"] OR [Abstract: "generative model"] OR [Title: "generative artificial intelligence"] OR [Abstract: "generative artificial intelligence"] OR [Title: "generative pre-trained transformer"] OR [Abstract: "generative pre-trained transformer"] OR [Title: "bidirectional encoder representations from transformers"] OR [Abstract: "bidirectional encoder representations from transformers"] OR [Title: "ai language model"] OR [Abstract: "ai language model"]] AND [[Title: medical practice] OR [Abstract: medical practice] OR [Title: healthcare] OR [Abstract: healthcare] OR [Title: clinical practice] OR [Abstract: clinical practice] OR [Title: medicine] OR [Abstract: medicine] OR [Title: medical treatment] OR [Abstract: medical treatment] OR [Title: patient] OR [Abstract: patient]] AND [E-Publication Date: (01/01/2022 TO 12/31/2023)]

5. IEEE Xplore
Year range 2022-2023
(("large language model" OR "LLM" OR "GPT" OR "BERT" OR "transformer model" OR "generative model" OR "generative AI" OR "generative artificial intelligence" OR "generative pre-trained transformer" OR "bidirectional encoder representations from transformers" OR "AI language model") AND (medical practice OR healthcare OR clinical practice OR medicine OR medical treatment OR patient)
