# Supplementary material for: Current applications and challenges in large language models for patient care: a systematic review
Source: Commun Med (Lond). 2025 Jan 21;5:26. doi: 10.1038/s43856-024-00717-2 (PMC11751060; doi:10.1038/s43856-024-00717-2)
Supplement: Supplementary file 2 — Description of Additional Supplementary Files [file 43856_2024_717_MOESM2_ESM.pdf]

Description of Additional Supplementary Files

File name: Supplementary Data 1

Description: Preferred Reporting Items for Systematic reviews and Meta-Analyses (PRISMA) checklist.

File name: Supplementary Data 2

Description: Excluded studies after full-text screening.

File name: Supplementary Data 3

Description: Overview of included studies and corresponding authors, year of publication, affiliation countries of authors, study design, medical specialty, purpose of study, large language model (LLM)/tool examined, target user, evaluation/setting, main outcome, and conclusion.

File name: Supplementary Data 4

Description: Evaluation of included studies according to the Mixed Methods Appraisal Tool (MMAT) 2018.

File name: Supplementary Data 5

Description: Taxonomy of identified LLM limitations, including their description, examples, and corresponding references.
